# *Gli1* Haploinsufficiency Leads to Decreased Bone Mass with an Uncoupling of Bone Metabolism in Adult Mice

**DOI:** 10.1371/journal.pone.0109597

**Published:** 2014-10-14

**Authors:** Yoshiaki Kitaura, Hironori Hojo, Yuske Komiyama, Tsuyoshi Takato, Ung-il Chung, Shinsuke Ohba

**Affiliations:** 1 Department of Sensory and Motor System Medicine, The University of Tokyo Graduate School of Medicine, Bunkyo-ku, Tokyo, Japan; 2 Division of Clinical Biotechnology, The University of Tokyo Graduate School of Medicine, Bunkyo-ku, Tokyo, Japan; 3 Department of Bioengineering, The University of Tokyo Graduate School of Engineering, Bunkyo-ku, Tokyo, Japan; Inserm U606 and University Paris Diderot, France

## Abstract

Hedgehog (Hh) signaling plays important roles in various development processes. This signaling is necessary for osteoblast formation during endochondral ossification. In contrast to the established roles of Hh signaling in embryonic bone formation, evidence of its roles in adult bone homeostasis is not complete. Here we report the involvement of *Gli1*, a transcriptional activator induced by Hh signaling activation, in postnatal bone homeostasis under physiological and pathological conditions. Skeletal analyses of *Gli1* ^+/−^ adult mice revealed that *Gli1* haploinsufficiency caused decreased bone mass with reduced bone formation and accelerated bone resorption, suggesting an uncoupling of bone metabolism. Hh-mediated osteoblast differentiation was largely impaired in cultures of *Gli1* ^+/−^ precursors, and the impairment was rescued by *Gli1* expression via adenoviral transduction. In addition, *Gli1* ^+/−^ precursors showed premature differentiation into osteocytes and increased ability to support osteoclastogenesis. When we compared fracture healing between wild-type and *Gli1* ^+/−^ adult mice, we found that the *Gli1* ^+/−^ mice exhibited impaired fracture healing with insufficient soft callus formation. These data suggest that *Gli1*, acting downstream of Hh signaling, contributes to adult bone metabolism, in which this molecule not only promotes osteoblast differentiation but also represses osteoblast maturation toward osteocytes to maintain normal bone homeostasis.

## Introduction

Hedgehog (Hh) signaling is a highly conserved pathway that plays important roles in various development processes. Hh signaling is indispensable for osteoblast formation in endochondral ossification, one of the two ossification processes in mammals, which forms bones in limbs, the trunk, and some head structures [1]. In this context, Indian hedgehog (Ihh) expressed in prehypertrophic chondrocytes is thought to act directly on progenitors in the perichondrium and the bone marrow to induce their differentiation into bone-forming osteoblasts [2]–[4]. The deletion of Ihh or smoothened (Smo), a transmembrane signaling transducer for Hh, caused no bone collar or primary spongiosa in mice [2], [4]. These mutant mice lacked the expression of runt-related transcription factor 2 (*Runx2*), a key determinant for osteoblasts [5], [6] and bone gamma-carboxyglutamate (gra) protein (osteocalcin; official gene symbol, *Bglap*), a bona fide marker for osteoblasts [1], in the perichondrium.

Gli transcription factors mediate the transcription of target genes downstream of Hh signaling. Gli1, a target gene of Hh signaling, acts as a transcriptional activator, whereas Gli2 and Gli3 can act as both activators and repressors [7]. The activator function of Gli2 and the repressor function of Gli3 were reported to mediate substantial aspects of the action of Ihh on bone development [8], [9]. In addition, we found that Gli1 participated in the Hh-mediated osteoblast formation collectively with Gli2 and Gli3 [10], [11].

Regarding postnatal roles of Hh signaling in bone metabolism, we reported that the haploinsufficiency of patched 1 (*Ptch1*), a transmembrane Hh receptor that represses signaling activity without Hh input, led to high bone mass in adult mice and humans [12]. Mice in which *Ptch1* was deleted in *Bglap*-positive mature osteoblasts showed low bone mass [13]. Although both of the mutants mentioned above had high bone turnover, where both osteoblastogenesis and osteoclastogenesis were enhanced, the balance shifted toward the opposite phenotypes. When an activator form of *Gli2* was forcibly expressed in osteoblast precursors expressing *Sp7* (osterix-Osx), another key determinant of osteoblasts, the skeleton was not affected at the fetal stage in mice. However, the mutants postnatally showed severe osteopenia with a decrease in osteoblast number, although the number of osteoclasts was not changed [14]. Thus, in contrast to the established roles of Hh signaling in embryonic bone formation, the evidence of its roles in adult bone homeostasis is not adequate. In particular, little is known about the roles of Gli1, a potent transcriptional activator induced by Hh signaling, in postnatal bone metabolism.

In the present study, we attempted to examine Hh signaling in the postnatal skeletal system, focusing on the functions of Gli1 in the bone metabolism. We analyzed skeletal phenotypes of *Gli1* heterozygous knockout mice postnatally *in vivo* and *in vitro*. We also compared fracture healing between mutant and wild-type (WT) mice. We report that *Gli1* haploinsufficiency affects adult bone metabolism, at least partly through the uncoupling of bone formation and bone resorption, leading to a decrease in bone mass and a delay in fracture healing in postnatal skeletons.

## Materials and Methods

### Animal Experiments

Wild-type C57BL/6J mice were obtained from Charles River Japan, and *Gli1* ^+/−^ mice were generated as previously described [15]. All experiments were performed in accord with the protocol approved by the Animal Care and Use Committee of The University of Tokyo (#KA13-5). Mice were kept in individual cages under controlled temperature and humidity with a 12-hr circadian rhythm. They were given *ad libitum* access to food and water. All efforts were made to minimize the suffering of the mice. Euthanasia of mice was performed with an overdose of barbiturates.

### Reagents and Vectors

Smoothened agonist (SAG) was purchased from Calbiochem (San Diego, CA; 566660). Receptor activator of NF-kappa-B ligand (RANKL) was purchased from Pepro Tech (Rocky Hill, NJ; 184-01791). Sonic hedgehog (Shh) was purchased from R&D Systems (Minneapolis, MN; 1845-SH). Cyclopamine was purchased from Enzo Life Sciences (Farmingdale, NY; BML-GR3334). Plasmids expressing human *GLI1* were constructed as previously described [12]. The adenoviral vector expressing human *GLI1-Biotin-3xFLAG-IRES-dsRed* was constructed using the pAd/PL-DEST vector and ViraPower Adnoviral Expression System (Life Technologies, Carlsbad, CA), according to the manufacturer's instructions. In brief, human *GLI1* cDNA carrying Biotin-3xFLAG tag [16] was initially cloned into pCID vectors [17]; *GLI1-Biotin-3xFLAG-IRES-dsRed* was then transferred into the pENTR1A vector in conjunction with the CAGGS promoter and subjected to adenoviral vector construction using the Gateway system. The pAd/PL-DEST expressing the CAGGS promoter-driven *GLI1-Biotin-3xFLAG-IRES-dsRed* was linearized with Pac I and transfected into 293A cells. After amplification, the virus was stored at −80°C. The viral titer was determined by an end-point titer assay using 293A cells.

### Cell Culture

C3H10T1/2 cells were obtained from the RIKEN Cell Bank (Ibaraki, Japan). Mouse primary bone marrow stromal cells were isolated from the long bones of 8-week-old male mice. Mouse primary osteoblast precursors were isolated from calvarias of mouse neonates. Cells were cultured in high-glucose Dulbecco's modified Eagle Medium (DMEM; Sigma-Aldrich, St. Louis, MO) containing 10% fetal bovine serum (FBS) and 1% penicillin/streptomycin. For osteogenic cultures, the cells were cultured in osteogenic media [18], [19] supplemented with Smoothened agonist (SAG). Co-culture experiments using mouse primary bone marrow stromal cells and bone marrow macrophages were performed as previously described [18]. The *in vitro* osteoclast differentiation of RAW264.7 cells was performed as previously described [20]. Adenoviruses expressing *GLI1-Biotin-3xFLAG-IRES-dsRed* (Ax-Gli1) or GFP (Ax-GFP) were used to infect cells at MOI (multiplicity of infection) 10. Alkaline phosphatase (ALP), von Kossa, and tartrate-resistant acid phosphatase (TRAP) staining were performed as previously described [12], [18].

### Real-Time RT-PCR

Total RNA extraction and real-time reverse transcription-polymerase chain reaction (RT-PCR) were performed as previously described [21]. All reactions were run in triplicate. The primer sequences are as follows: *β-actin*, AGATGTGGATCAGCAAGCAG (forward) and GCGCAAGTTAGGTTTTGTCA (reverse); *Alp*, GCTGATCATTCCCACGTTTT (forward) and CTGGGCCTGGTAGTTGTTGT (reverse); *Ibsp*, CAGAGGAGGCAAGCGTCACT (forward) and CTGTCTGGGTGCCAACACTG (reverse); *Bglap*, AAGCAGGAGGGCAATAAGGT (forward) and TTTGTAGGCGGTCTTCAAGC (reverse); *Gli1*, GCACCACATCAACAGTGAGC (forward) and GCGTCTTGAGGTTTTCAAGG (reverse); *Gli2*, CTGAAGGATTCCTGCTCGTG (forward) and ACAGTGTAGGCCGAGCTCAT (reverse); *Runx2*, CCGCACGACAACCGCACCAT (forward) and CGCTCCGGCCCACAAATCTC (reverse); *Sp7*, ACTCATCCCTATGGCTCGTG (forward) and GGTAGGGAGCTGGGTTAAGG (reverse); *Tnfsf11*, AGCCATTTGCACACCTCAC (forward) and CGTGGTACCAAGAGGACAGAGT (reverse); *Tbfrsf11b*, GTTTCCCGAGGACCACAAT (forward) and CCATTCAATGATGTCCAGGAG (reverse); *Dmp1*, CAGTGAGGATGAGGCAGACA (forward) and TCGATCGCTCCTGGTACTCT (reverse); *Sost*, AAGCCGGTCACCGAGTTGGT (forward) and GTGAGGCGCTTGCACTTGCA (reverse); *Ptch1*, CTGGACTCTGGCTCCTTGTC (forward) and CAACAGTCACCGAAGCAGAA (reverse); *Ctsk*, ACGGAGGCATCGACTCTGAA (forward) and GATGCCAAGCTTGCGTCGAT (reverse); and *Nfatc1*, CCTTCGGAAGGGTGCCTTTT (forward) and AGGCGTGGGGCCTCAGCAGG (reverse).

### Luciferase Assay

Cells were plated onto 24-well plates and transfected with 0.4 µg of DNA in a mixture containing the reporter plasmids (8×3′-Gli BS-luc) [22], the control reporter plasmids encoding Renilla luciferase, and effector plasmids (pCMV-*myc-GLI1*) or adenoviral vectors expressing *GLI1*. A dual-luciferase assay was performed as previously described [12].

### Immunoblot

Whole-cell lysates were isolated using RIPA buffer as previously described [23]. Sodium dodecyl sulfate-polyacrylamide gel electrophoresis (SDS-PAGE) and immunoblotting were performed using anti-Gli1 (sc-20687, 1∶1000, Santa Cruz Biotechnology, Santa Cruz, CA) and HRP-conjugated anti-FLAG M2 antibodies (A8592-2MG, 1∶500, Sigma-Aldrich) as previously described [12].

### Radiological analysis

X-ray photographs of the left tibiae of WT and *Gli1* ^+/−^ mice (n = 10 each) were taken using a soft x-ray system (M-60; Softex Co., Tokyo). Micro-computed tomography (CT) scanning of the harvested femurs was performed using a microfocus X-ray CT system SMX-90CT (Shimadzu, Kyoto, Japan) under the following conditions: tube voltage, 90 kV; tube current, 110 µA; layer thickness, 5.3 mm; and field of view (XY), 10.4 mm. The resolution of one CT slice was 512×512 pixels. The three-dimensional construction software package TRI/3D-BON (Ratoc System Engineering, Tokyo) was used for quantitative analysis.

### Histological analysis

We intraperitoneally injected calcein (0.16 mg per 10 g of body weight; Sigma) into mice 4 days and 1 day before sacrifice. We stained the undecalcified sections of the femur with toluidine blue, von Kossa, and TRAP as previously described [12]. Images were taken using an Axio Imager A1 (Carl Zeiss, Jena, Germany) and processed using AxioVision (Carl Zeiss). Histomorphometric analyses were performed using the HistometryRT CAMERA system. We analyzed five mice for each group.

### Fracture model

Ten 8-week-old male mice were used in each group. Under general anesthesia with isofluorane in O_2_, the left hind limb was shaved and sterilized for surgery. A 15-mm incision was made longitudinally, and a blunt dissection of the muscle was made to expose the tibia. A transverse osteotomy was performed using disk-shaped dental steel bars at the mid point of the tibia. The fracture was repositioned, and then the full-length of the bone marrow cavity was internally stabilized as previously described [24]. After irrigation with saline, the skin was closed with 4-0 nylon sutures. Fourteen days after surgery, the tibias were harvested from the euthanized mice.

### Statistical analysis

The means of groups were compared by an analysis of variance (ANOVA), and the significances of the differences were determined by Student's t-test. P-values <0.05 were considered significant.

## Results

### *Gli1* haploinsufficiency causes decreased bone mass in adult mice

As reported, we found that *Gli1* ^−/−^ mice had no gross abnormalities at birth [11], [15]. Although *Gli1* ^−/−^ pups were born in Mendelian ratios, the number of *Gli1* ^−/−^ adults turned out to be approximately 10% of the total pups obtained because of their reduced survival rates during the first 10 days after birth (Table 1). In addition, the growth rate of the surviving *Gli1* ^−/−^ mice was significantly lower than those of both the wild-type (WT) or *Gli1* ^+/−^ mice, and the body weights of the 8-week-old *Gli1* ^−/−^ mice were approximately 20% lower than those of the WT and *Gli1* ^+/−^ mice (Figure S1).

**Table 1:** Survival rate of *Gli1* mutant mice during postnatal 10 days.

Genotype	Postnatal day	
	1	10
*Gli1* ^+/+^	11 (30.6)	37 (28.5)
*Gli1* ^+/−^	16 (44.4)	81 (62.3)
*Gli1* ^−/−^	9 (25.0)	12 (9.2)
Total	36	130

Percentages are in parentheses.

**Figure 1:**
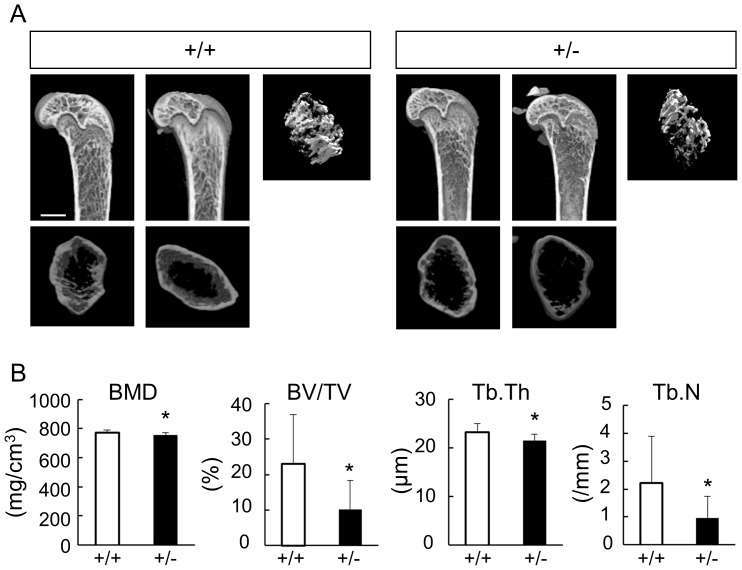
Radiological findings of long bones in wild-type (WT) and *Gli1* ^+/−^ mice. (**A**) Three-dimensional micro-computed tomography (3D-micro-CT) images of the distal femurs of representative 8-week-old WT and *Gli1* ^+/−^ male mice. Sagittal sections, transverse sections, and 3D reconstruction images of the primary spongiosa are shown for each genotype. Bar, 1 mm. (**B**) Histomorphometric analyses of 3D-micro-CT data. BMD, bone mineral density; BV/TV, bone volume per tissue volume; Tb.Th, trabecular thickness; Tb.N trabecular number parameters. Data are means ± SDs of eight male mice per genotype. *p<0.05 vs. WT.

These findings suggest that the *Gli1* ^−/−^ mice were largely affected by systemic abnormalities due to complete loss of *Gli1* gene. Therefore, to investigate the roles of Gli1 in adult bone metabolism as directly as possible, we analyzed the skeletal system in *Gli1* ^+/−^ male mice, which had weights and gross appearance comparable to those of the WT male mice. Micro-computed tomography (µ-CT) analyses of distal femurs revealed that the trabecular density of the 8-week-old *Gli1* ^+/−^ mice was reduced compared to that of WT mice (Figure 1A). The bone morphometric analysis using the micro-CT data supported the finding, as the *Gli1* ^+/−^ mice showed less bone mineral density (BMD) along with decreased parameters for bone formation (Figure 1B). These skeletal phenotypes were also observed in the *Gli1* ^+/−^ female mice (Figure S2). In contrast, there was no significant difference in the cortical bone between WT and *Gli1* ^+/−^ mice (Figure S3), suggesting differences between trabecular and cortical bones with regard to the contribution of Gli1.

**Figure 2:**
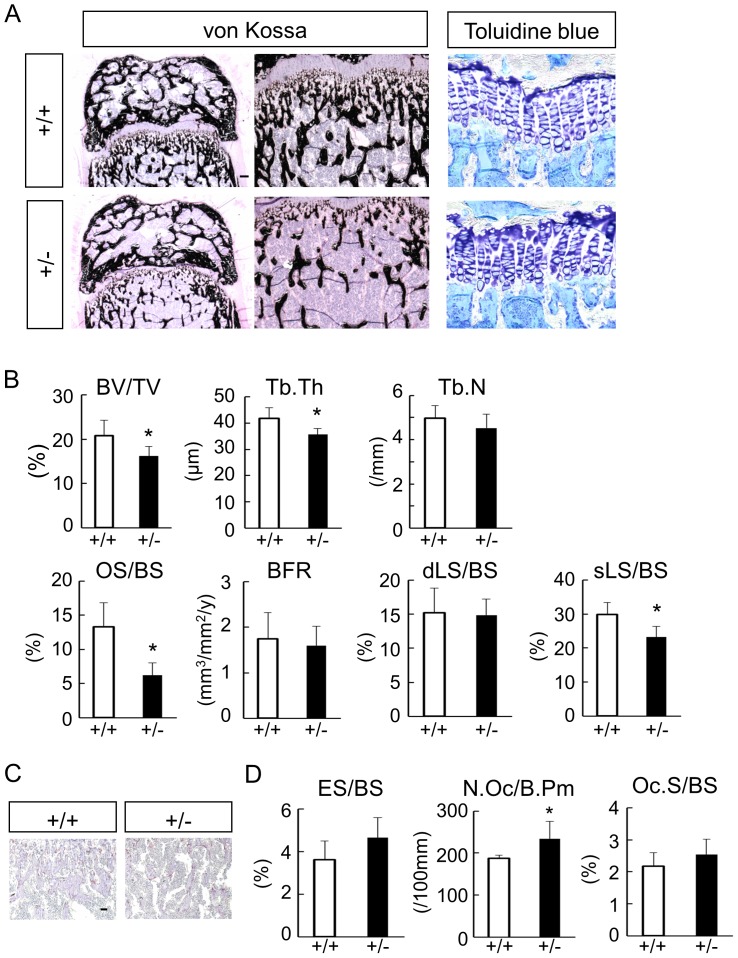
Histological findings of adult WT and *Gli1* ^+/−^ mice. (**A**) von Kossa staining and toluidine blue staining of the distal femur sections of representative 8-week-old WT and *Gli1* ^+/−^ male mice. Bar, 100 µm. (**B**) Histomorphometric analyses of bone volume and bone formation parameters in distal femurs from 8-week-old WT and *Gli1* ^+/−^ male mice. OS/BS, osteoid surface per bone surface; MAR, mineral apposition rate; BFR, bone formation rate per bone surface; dLS/BS, double-labeled surface per bone surface; sLS/BS, single-labeled surface per bone surface. Data are means ± SDs of five male mice per genotype. *p<0.05 vs. WT. (**C**) TRAP staining of the distal femur sections of representative 8-week-old WT and *Gli1* ^+/−^ male mice. Bar, 100 µm. (**D**) Histomorphometric analyses of bone resorption parameters in the distal femurs of 8-week-old WT and Gli1^+/−^ male mice. ES/BS, eroded surface per bone surface; N. Oc/B. Pm, number of osteoclasts per 100 mm of bone perimeter; Oc. S/BS, osteoclast surface per bone surface. In (**B**) and (**D**), data are means ± SDs of five mice per genotype. *p<0.05 vs. WT.

The histological analyses of the distal femurs of 8-week-old mice showed that *Gli1* ^+/−^ mice had reduced trabecular bones compared to the WT mice (Figure 2A, see von Kossa), whereas the growth plate appeared normal (Figure 2A, see toluidine blue). Indeed, the bone volume/tissue volume (BV/TV) and trabecular thickness (Tb.Th) values were significantly decreased in *Gli1* ^+/−^ mice compared to WT mice, as observed in micro-CT-based analyses (Figure 2B). *Gli1* ^+/−^ mice also had significantly lower values of osteoid surface/bone surface (OS/BS) and single-labeled surface/bone surface (sLS/BS), which are parameters of the osteogenic capacity (Figure 2B).

Regarding bone resorption, the numbers of TRAP-positive osteoclasts were significantly higher in the *Gli1* ^+/−^ mice compared to the WT mice (Figure 2C and D, see N.Oc/B.Pm). Consistent with this finding, the *Gli1* ^+/−^ mice showed a trend toward increased bone resorption capacity parameters, compared to WT mice (Figure 2D, see ES/BS and Oc.S/BS). These data suggest that *Gli1* haploinsufficiency causes an uncoupling of bone turnover in adult mice, which leads to decreased bone mass.

### Osteoblast differentiation is impaired by *Gli1* haploinsufficiency

**Figure 3:**
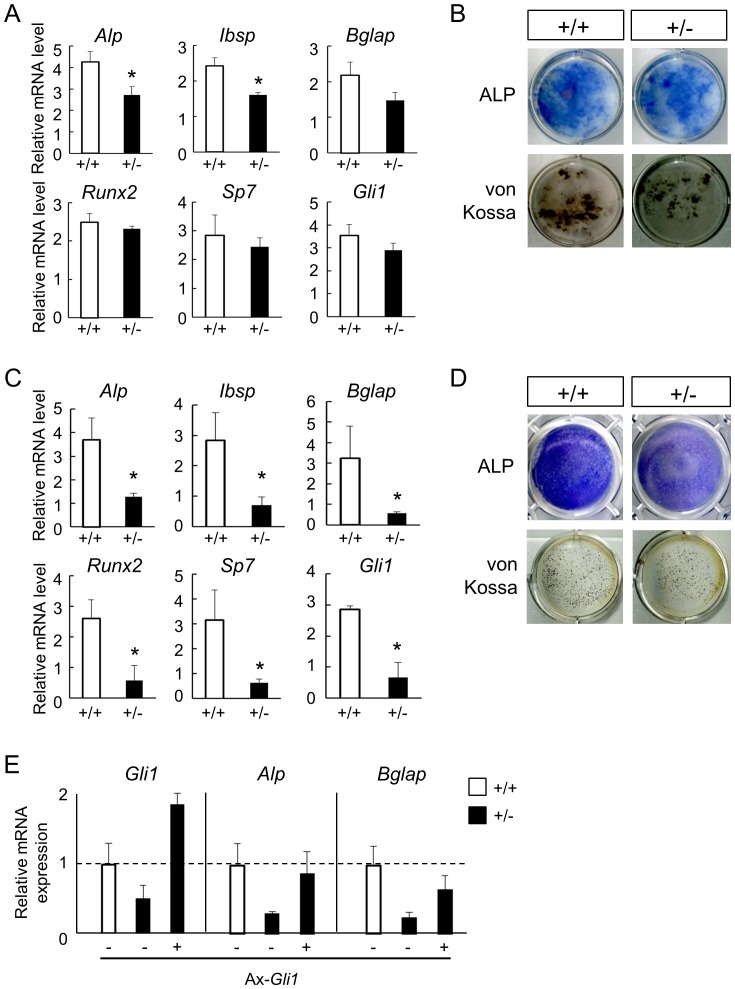
Osteoblast differentiation in cultures of precursor cells from WT and *Gli1* ^+/−^ mice. (**A**) mRNA expression of osteoblast marker genes in 14-day osteogenic cultures of BMSCs in the presence of the Smoothened agonist. mRNA expression was analyzed by real-time RT-PCR. (**B**) ALP and von Kossa stainings in 14-day osteogenic cultures of BMSCs in the presence of the Smoothened agonist. (**C**) mRNA expression of osteoblast marker genes in 7-day osteogenic cultures of osteoblast precursors (OPs) isolated from neonatal calvarias, in the presence of the Smoothened agonist. (**D**) ALP and von Kossa staining in 7-day osteogenic cultures of OPs in the presence of the Smoothened agonist. (**E**) Rescue of the expression levels of osteoblast marker genes in *Gli1* ^+/−^ OPs by the adenoviral overexpression of *Gli1* in the presence of the Smoothened agonist. WT or *Gli1* ^+/−^ OPs were infected with Ax-GFP (−) or Ax-Gli1 (+) at MOI 10.

To investigate whether *Gli1* haploinsufficiency caused decreased osteogenic capacity through the impairment of osteoblast differentiation from precursors, we examined osteoblast differentiation in *ex vivo* cultures of primary bone marrow stromal cells (BMSCs) harvested from 8-week-old WT and *Gli1* ^+/−^ mice (Figure 3A and B) and primary osteoblast precursors (OPs) from neonates of each genotype (Figure 3C and D). Given that *Gli1* expression is induced upon Hh signaling, we suspected that any difference resulting from the loss of one allele of *Gli1* would be observed under Hh signaling-activated conditions. We therefore performed the experiments in the presence of Smoothened agonist (SAG), a small molecule that activates Hh signaling. The mRNA expression levels of osteoblast-related genes were lower in *Gli1* ^+/−^ BMSCs than in WT BMSCs. In particular, alkaline phosphatase (*Alp*) and integrin-binding sialoprotein (*Ibsp*) expression levels were significantly reduced in the *Gli1* ^+/−^ BMSCs (Figure 3A), which was consistent with our previous finding that Gli1 directly induced the expression of these two genes [11]. ALP activity and matrix calcification, key features of osteoblasts, were also suppressed in the *Gli1* ^+/−^ BMSCs as evidenced by ALP and von Kossa staining (Figure 3B). The impaired osteoblast differentiation due to loss of one allele of *Gli1* was more prominent in the OPs. All of the genes tested here showed significantly lower expression levels in the *Gli1* ^+/−^ OPs compared to WT (Figure. 3C), and ALP activity and calcification were impaired in the *Gli1* ^+/−^ OPs (Figure 3D). Lastly, *Gli1* was reduced via the loss of one allele of *Gli1* in both cell types, although a significant reduction was observed only in the OPs (Figure 3A and C, see *Gli1*).

The data above suggest that *Gli1* haploinsufficiency affects osteoblast differentiation in a cell-autonomous manner. We then attempted to further verify the hypothesis by testing whether the recovery of *Gli1* expression would rescue the osteoblast phenotypes in *Gli1* ^+/−^ cells. We prepared an adenoviral vector expressing *Gli1* (Ax-*Gli1*) (Figure S4A). Gli1 protein expression induced by Ax-*Gli1* and its function were confirmed by western blotting using a specific antibody, luciferase reporter assays using the Gli-responsive element, and mRNA expression analyses of *Alp* and *Ibsp* in C3H10T1/2 cells (Figures S4B–D). As shown in Figure 3E, the introduction of *Gli1* into *Gli1* ^+/−^ OPs induced the expression of *Alp*, an early osteoblast marker, at a level comparable to that in WT OPs. The expression of *Bglap*, an osteoblast marker that is expressed at a later stage, was also upregulated but not fully recovered by *Gli1* introduction. Thus, *Gli1* haploinsufficiency in osteoblast precursors is likely to cause impairment of their differentiation, which underlies reduced bone formation in *Gli1* ^+/−^ mice.

### The Hh-Gli1 axis is involved in the capacity of osteoblasts/osteocytes to support osteoclastogenesis, but not in osteoclastogenesis itself

Bone homeostasis is achieved by the coupling of bone formation and bone resorption via cross-talk between osteoblasts/osteocytes and osteoclasts. Mature osteoblasts and osteocytes are known to support osteoclastogenesis by expressing the receptor activator of NF-kappa-B ligand (RANKL; official symbol, *Tnfsf11* - tumor necrosis factor (ligand) superfamily, member 11) and osteoprotegerin (OPG; official symbol, *Tnfrsf11b* - tumor necrosis factor receptor superfamily, member 11b), a stimulator and an inhibitor of osteoclastogenesis, respectively [25]–[28]. However, *Gli1* ^+/−^ mice exhibited impaired bone formation and accelerated bone resorption, suggesting an uncoupling state of bone metabolism.

**Figure 4:**
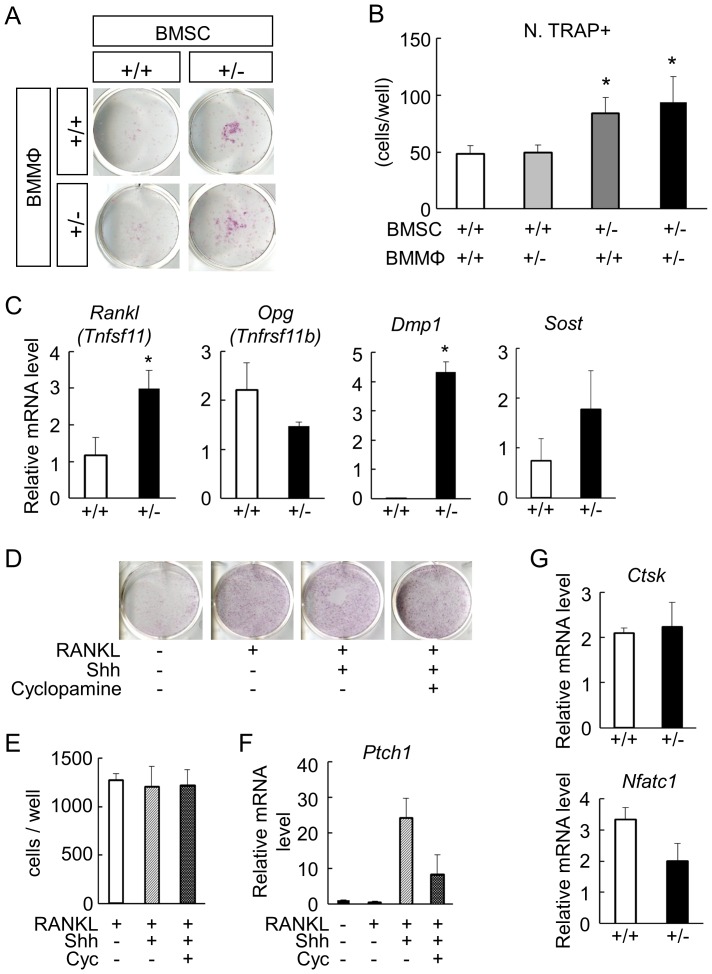
Osteoclast differentiation in cultures of bone marrow cells from WT and *Gli1* ^+/−^ mice. (**A**) Formation of TRAP-positive multinucleated osteoclasts by the co-culture of BMSCs and BMMΦ derived from WT or *Gli1* ^+/−^ mice. (**B**) The numbers of osteoclasts expressed as means ± SDs of 4 wells per group in (A). *p<0.05 vs. the control group (WT BMSC × WT BMMΦ). (**C**) mRNA expression of *Rankl*, *Opg*, *Dmp1*, and *Sost* in 14-day osteogenic cultures of BMSCs. The mRNA expression was analyzed by real-time RT-PCR. *Rankl*, receptor activator of nuclear factor-κB ligand; *Opg*, osteoprotegerin; *Dmp1*, dentin matrix acidic phosphoprotein 1; *Sost*, sclerostin. *p<0.05 vs. WT. (**D**) Formation of TRAP-positive multinucleated osteoclasts in 5-day cultures of RAW cells in the presence or absence of sonic hedgehog (Shh) and cyclopamine (Cyc). (**E**) The numbers of osteoclasts in (D) expressed as means ± SDs of 4 wells per group. (**F**) mRNA expression of *Ptch1* in cultured RAW cells in the presence or absence of Shh and cyclopamine (Cyc). Data are means ± SDs of 4 wells per group. (**G**) mRNA expression of cathepsin K (*Ctsk*) and *NFATc1* in osteoclasts derived from WT or *Gli1* ^+/−^ BMMΦ. Cells were cultured with recombinant M-CSF (10 ng/mL), RANKL (100 ng/mL), and Shh (25 nM). The mRNA expression was analyzed by real-time RT-PCR.

To identify the cellular mechanism underlying the aberrant state in *Gli1* ^+/−^ bones, we conducted co-culture experiments using BMSC and bone marrow macrophages (BMMΦ) derived from either WT or *Gli1* ^+/−^ mice. The number of TRAP-positive multinucleated osteoclasts co-cultured with *Gli1* ^+/−^ BMMCs was significantly higher than those with WT BMMCs, regardless of the *Gli1* genotypes in the BMMΦ (Figure 4A and B). Thus, bone-forming cells and their progenitors, not osteoclastic cells, were likely to be responsible for the abnormalities in osteoclastogenesis of the *Gli1* ^+/−^ mice. This led us to analyze BMSCs in terms of their ability to support the osteoclastogenesis.

In BMSCs harvested from the femurs of 8-week-old *Gli1* ^+/−^ mice, the mRNA expression of RANKL (*Tnfsf11*) was significantly upregulated, whereas that of OPG (*Tnfrsf11b*) showed a trend toward downregulation compared to WT BMSCs (Figure 4C). In addition, expression levels of dentin matrix acidic phosphoprotein 1 (*Dmp1*) and sclerostin (*Sost*), markers for osteocytes, were higher in the *Gli1* ^+/−^ BMSCs than in the WT (Figure 4C). This trend was also observed in OPs (Figure S5). Taken together with the downregulation of osteoblast marker genes in *Gli1* ^+/−^ BMSCs, these findings indicate that *Gli1* haploinsufficiency may promote a premature differentiation of osteoblast precursors into osteocytes, which have been reported as a major source of RANKL [25], and these results may indicate that the premature differentiation not only affects bone formation, but also stimulates bone resorption by inducing RANKL expression at a supraphysiological level. Given that the recovery of *Gli1* expression negated the upregulation of RANKL mRNA expression in *Gli1* ^+/−^ BMSCs (Figure S6), it is also possible that *Gli1* not only suppresses the differentiation of osteoblast precursors into osteocytes, but also negatively acts on the transcription of RANKL.

We next investigated the cell-autonomous effects of Hh signaling on osteoclastogenesis using RAW264.7 cells, which have been shown to differentiate into TRAP-positive osteoclasts in the presence of RANKL [20], [29]. Treatment with neither Shh nor cyclopamine, an inhibitor of hedgehog signaling, affected the RANKL-induced osteoclast differentiation of RAW264.7 cells (Figures 4D and E) although the cells were responsive to Hh signaling, as indicated by the expression change of *Ptch1*, a readout of the Hh signaling, upon Shh and cyclopamine (Figure 4F). Finally, we analyzed osteoclastogenesis of primary BMMΦ harvested from 8-week-old WT or *Gli1* ^+/−^ mice. Expression levels of cathepsin K (*Ctsk*) and *Nfatc1*, markers for osteoclasts, were not affected by loss of *Gli1* in cultures of BMMΦ with M-CSF and RANKL (Figure 4G).

Thus, the Hh-Gli1 axis is unlikely to mediate osteoclastogenesis in a cell-autonomous manner, which further suggests that the accelerated bone resorption in *Gli1* ^+/−^ mice is not caused by defects in osteoclastic cells. Abnormalities in osteoblast precursors due to *Gli1* haploinsufficiency may mediate all the aspects of the disruption of bone homeostasis in *Gli1* ^+/−^ bones, which explains why *Gli1* ^+/−^ mice have a low bone mass phenotype.

### Fracture healing was impaired by *Gli1* haploinsufficiency

**Figure 5:**
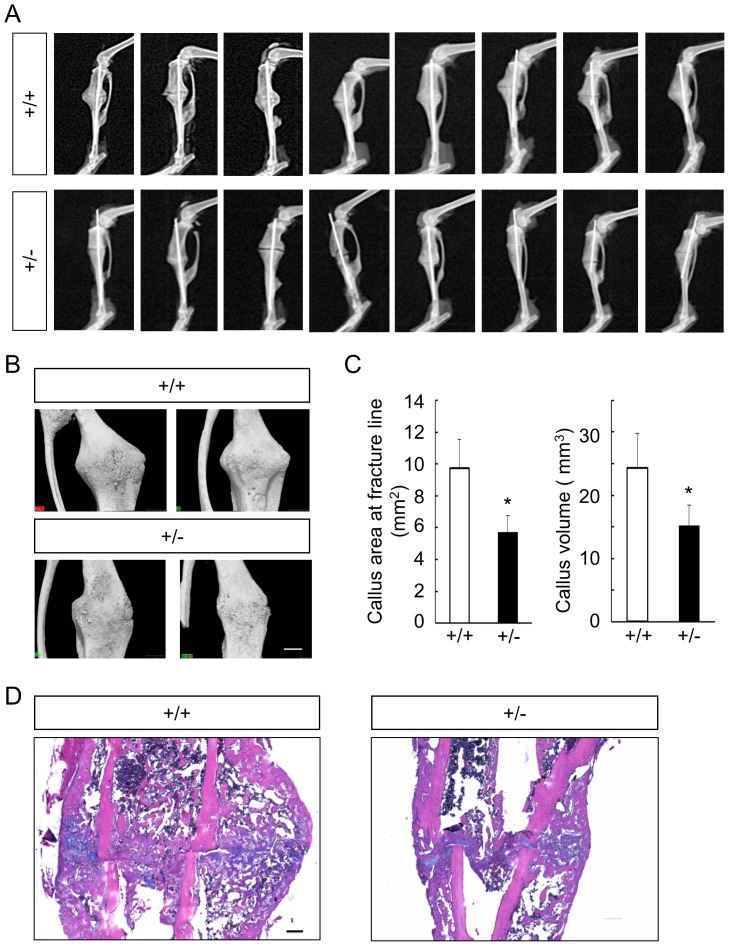
Comparison of bone fracture healing between 8-week-old WT and *Gli1* ^+/−^ mice. (**A**) Soft X-ray pictures of the fracture sites of all WT (n = 8) and *Gli1* ^+/−^ (n = 8) male mice tested at 2 weeks after the fracture. (**B**) Representative micro-CT images of the callus in WT and *Gli1* ^+/−^ male mice. Bar, 1 mm. (**C**) The areas of horizontal cross-sections at fracture lines (left) and the volume of the calluses (right) of WT and *Gli1* ^+/−^ mice, calculated using 3D-micro-CT data. Data are means ± SDs of eight mice per genotype. *p<0.05 vs. WT. (**D**) H&E and alcian blue double staining of the calluses 2 weeks after the fracture. Bar, 200 µm.

We next set out to examine the involvement of *Gli1* in postnatal bones under a pathological condition, comparing fracture healing between WT and *Gli1* ^+/−^ mice in a model that was surgically created in the tibias of 8-week-old males. During the fracture healing process, both intramembranous ossification and endochondral ossification are observed, and osteo-chondroprogenitor cells from the periosteum adjacent to fracture sites are major source of cells that contribute to the healing process [30], [31]. Fracture healing was evaluated 2 weeks after the surgery, given that bony bridging at the fracture site was typically observed as early as the point [30], [31]. In soft X-ray analyses, we observed impairment of callus formation and bone union in *Gli1* ^+/−^ mice compared to WT mice, as well as the variability and reproducibility of the fracture model itself (Figure 5A). Using 3D-micro-CT analyses, we evaluated the areas of the calluses on horizontal cross-sections at the fracture lines and the volume of the callus. Both the areas and the volumes of the callus in the *Gli1* ^+/−^ mice were significantly lower than those in WT mice (Figures 5B and C).

We then performed histological analyses on sections stained with the hematoxylin-eosin (H–E) and alcian blue to identify differences in the healing process between WT and *Gli1* ^+/−^ mice (Figure 5D). In WT mice, large volumes of soft callus surrounded by hard calluses were observed around the fracture sites, as previously reported [32] (Figure 5D). However, in *Gli1* ^+/−^ mice, both the soft and hard calluses were reduced compared to those in the WT mice (Figure 5D), suggesting that both endochondral ossification and intramembranous ossification were affected by *Gli1* haploinsufficiency during the fracture healing process.

## Discussion

The present study had six major findings. (1) Adult bone mass was affected by *Gli1* haploinsufficiency in mice, although body length and body weight were not. (2) The low bone mass phenotypes were accompanied by impaired bone formation and accelerated bone resorption, that is, an uncoupling of bone metabolism. (3) *Gli1* haploinsufficiency had a negative impact on Hh-mediated osteoblast differentiation in cultures of precursors. (4) Despite the impairment of osteoblast differentiation, the expression levels of *Dmp1*and *Rankl* were upregulated in cultures of *Gli1* ^+/−^ precursors, suggesting that *Gli1* haploinsufficiency induced the premature differentiation of osteoblasts into osteocytes, which have a greater ability to promote osteoclastogenesis than do osteoblasts. (5) Hh-Gli1 was not involved in osteoclastogenesis in a cell-autonomous manner in vitro. (6) *Gli1* haploinsufficiency affected fracture healing in adult mice. Based on these findings, we propose that Gli1, acting downstream of Hh signaling, not only promotes osteoblast differentiation but also acts as a repressor of osteoblast maturation toward osteocytes to maintain normal bone homeostasis in adult mice.

There are two possible mechanisms underlying the aberrant upregulation of osteocyte marker genes in *Gli1* ^+/−^ precursors. The first possibility is that the overall differentiation of osteoblast precursors is accelerated by *Gli1* haploinsufficiency, although the differentiation program is kept normal. The second possibility is that the program itself is disturbed by *Gli1* haploinsufficiency, and the disturbance may induce the premature differentiation of precursors into osteocytes by skipping proper phases of osteoblast differentiation. The second possibility is more likely, because osteocyte marker genes (*Dmp1* and *Sost*) were highly induced in *Gli1* ^+/−^ cells, despite downregulation of both early and late osteoblast marker genes (*Alp*, *Ibsp*, *Runx2*, *Sp7*, and *Bglap*). If the first possibility was the case, *Gli1* ^+/−^ precursors would show upregulation of both osteoblast and osteocyte marker genes.

It remains to be elucidated how *Gli1* haploinsufficiency disturbs the osteoblast differentiation program, which results in accelerated bone resorption as well as decreased bone formation, i.e., an uncoupling of bone metabolism in development. *Gli1*, collectively with *Gli2* and *Gli3*, is involved in the specification of osteo-chondroprogenitor cells in the perichondrium into an osteoblast lineage, and it promotes early osteoblast differentiation in a Runx2-independent manner [11]. The removal of *Smo* after the specification of *Sp7*-positive osteoblast precursors showed normal osteoblast development, suggesting that Hh signaling is not required for the differentiation of *Sp7*-positive osteoblast precursors into osteoblasts [33]. In contrast, the phenotypes of *Gli1* ^+/−^ mice suggest that Gli1 acts to repress the maturation of osteoblasts into osteocytes in postnatal bones directly or indirectly via a negative feedback loop-like mechanism, in addition to its specifier function. In addition to the repressive effects of Gli1 on the terminal differentiation of osteoblasts, Gli1-mediated negative regulation of *Rankl* transcription may also explain the enhanced osteoclastogenesis in *Gli1* ^+/−^ mice. An extensive search for Gli binding regions in the osteoblast genome will be useful for understanding the precise roles of Gli1 in bone metabolism.

Given that one copy of *Gli1* allele was removed in all the cells of the mice used in the present study, cells other than skeletal lineages may also be involved in the osteoblast phenotypes seen in the mutants via systemic factors or direct contact with osteoblastic cells. Indeed, the body weights of the 8-week-old *Gli1* ^−/−^ mice were about 20% less than those of the WT and *Gli1* ^+/−^ mice, suggesting systemic abnormalities upon complete loss of *Gli1*. Stage- and tissue-specific manipulation of *Gli1* using a floxed *Gli1* allele would clarify the distinct function of *Gli1* at different stages of osteoblast development, although such mutant mice are not yet available.

The involvement of Hh signaling in the regulation of bone metabolism has been debated. We previously found that *Ptch1* ^+/−^ mice and patients with nevoid basal cell carcinoma syndrome, in which Hh signaling was activated by the loss of one copy of the *Ptch1* allele, demonstrated high bone mass phenotypes [12], whereas Mak et al. reported that the disruption of the gene in *Bglap*-positive cells caused low bone mass [13]. At cellular levels, a common phenomenon underlies the contradictory phenotypes. Both osteoblastogenesis and osteoclastogenesis were enhanced in both mutants. Therefore, those different outcomes might be due to an alteration of the balance between enhanced bone formation and bone resorption. These studies and the present investigation have provided consistent evidence of the direct promotion of osteoblastogenesis by Hh signaling in adult mice.

In contrast, there are some arguments with respect to the involvement of Hh signaling in osteoclastogenesis. The studies mentioned above [12], [13] and the present one support the concept that Hh signaling stimulates osteoclastogenesis indirectly via the augmentation of osteoblasts or osteocytes. Conversely, Heller et al. described that the inhibition of Smo suppressed osteoclastogenesis in a cell-autonomous manner [34]. The reasons for these conflicting findings should be elucidated with regard to cell type and culture conditions. Joeng et al. recently reported postnatal skeletal phenotypes in mice expressing a constitutively active form of *Gli2* in *Sp7*-positive cells. The mutants showed osteopenia with decreased bone formation and unaltered bone resorption, although osteoblast differentiation was enhanced in cultures of precursor cells isolated from the mutants [14]. Although *Gli1* is thought to be upregulated in the mutants as *Ptch1* upregulation was confirmed [14], their skeletal phenotypes are seemingly inconsistent with those in the *Gli1* ^+/−^ mice. The discrepancy may be caused by the difference in populations in which Hh signaling was manipulated or the difference in Gli factors that were manipulated between the studies. Overall, these results suggest that as Joeng et al. mentioned [14], the roles of Hh signaling in bone metabolism depend on its target population, the timing of its activation, and complex regulation by Gli factors downstream of the signaling; the roles are possibly modulated by non-skeletal cells or factors.

We demonstrated roles of Gli1 in the fracture healing process as well as in mouse adult bone homeostasis. A previous report on defects of bone healing in *Smo*-deleted mice [35] supports the impairment of callus formation in the *Gli1* ^+/−^ mice although the tested models are different between that study and our present investigation. Given that cartilage development was not largely affected by the complete loss of *Gli1* [11], the reduced size of soft calluses during fracture healing of the *Gli1* ^+/−^ mice was unexpected. In development, Gli3 repressor, rather than Gli activators, was shown to play a major role in the Hh-mediated control of cartilage formation [8]. Thus, it is possible that the involvement of *Gli1* in chondrogenesis and/or cartilage metabolism is different between embryos and adults or physiological conditions and pathological ones. Hh signaling may require a contribution from the Gli activators during fracture healing, where the rapid growth of cartilaginous tissues is likely to depend more on cellular proliferation than on increases in cellular volume or matrix deposition [32]. The promotion of proliferation is known to be a direct action of Hh signaling on chondrocytes [36].

When discussing the regulation of the adult skeleton by Hh-Gli signaling under physiological and pathological conditions, we should consider target cell types and sources of Hh ligands. Maeda et al. demonstrated that Ihh secreted from chondrocytes in the growth plate is required for the maintenance of trabecular bones in postnatal mice [37]. Type I collagen-positive cells in bone lining were shown to express Ihh in humans [38]. Both Ihh expressed in soft calluses [39] and sonic hedgehog (Shh) expressed in the periosteum [40] or osteoblasts/osteocytes [41] have been implicated in fracture healing. These findings imply that Gli1 is likely to contribute to adult bone metabolism upon the inputs of Ihh and Shh. In addition, given that Hh signaling acts to maintain the skeleton in adults, and some species close the growth plate after puberty, skeletal components other than growth plate chondrocytes may produce Hh ligands in this context.

In conclusion, the results of the present study lead us to propose the involvement of *Gli1* in postnatal bone homeostasis under physiological and pathological conditions although further studies are necessary to obtain an integrative understanding of the roles of all Gli family members in this context. Collectively with previous studies, the present study also indicates the importance of tissue- and stage-specific manipulation of Hh signaling for the treatment of bone-related disease, as well as the need for a greater understanding of all the actions of Hh signaling on the skeletal tissue throughout life.

## Supporting Information

Figure S1 **Gross appearance of WT and** ***Gli1*** **mutant male mice.** (**A**) Gross appearance of WT, *Gli1* ^+/−^, and *Gli1* ^−/−^ male mice at 8 weeks of age. (**B**) Comparison of postnatal growth between WT, *Gli1* ^+/−^, and *Gli1* ^−/−^ male mice. Body weight was measured on the indicated dates after birth. *p<0.05 vs. WT or *Gli1* ^+/−^.
(PDF)

Figure S2 **Radiological analyses of long bones in female WT and** ***Gli1*** **^+/−^ mice.** (**A**) 3D-micro-CT images of the distal femurs of representative 8-week-old WT and *Gli1* ^+/−^ female mice. Sagittal sections, transverse sections, and 3D reconstruction images of the primary spongiosa are shown for each genotype. Bar, 1 mm. (**B**) Histomorphometric analyses of the 3D-micro-CT data in (A). BMD, bone mineral density; BV/TV, bone volume per tissue volume; Tb.Th, trabecular thickness; Tb.N, trabecular number parameters. Data are means ± SDs of five female mice per genotype. *p<0.05 vs. WT.
(PDF)

Figure S3 **Radiological analyses of cortical bones in WT and** ***Gli1*** **^+/−^ mice.** (**A**) 3D-micro-CT images of distal femurs of representative 8-week-old WT and *Gli1* ^+/−^ male mice. Transverse sections of the primary spongiosa are shown for each genotype. Bar, 500 µm. (**B**) Histomorphometric analyses of 3D-micro-CT data in (A). Cv/Av, cortical bone volume per all bone volume; Cvt, cortical bone thickness; BMD, bone mineral density. Data are means ± SDs of ten male mice per genotype.
(PDF)

Figure S4 **Construction of adenoviral vector expressing** ***GLI1*** **.** (**A**) Schematic representation of pAd/PL-DEST vectors expressing *GLI1*. (**B**) Luciferase reporter assay using the 8×3′-Gli BS-luc in combination with *dsRed*, *Myc-GLI1*, and the constructed adenoviral vector (*GLI1-IRES-deRed*). The luciferase assay was performed 48 hours after transfection in C3H10T1/2 cells. (**C**) Protein expression of GLI1 in C3H10T1/2 cells transfected with *Myc-GLI1* or adenovirally transduced with *GLI1-IRES-dsRed*. (**D**) mRNA expression of *Alp* and *Ibsp* in C3H10T1/2 cells transfected with *Myc-GLI1* or adenovirally transduced with *GLI1-IRES-dsRed*.
(PDF)

Figure S5 **mRNA expression of** ***Rankl*** **,** ***Opg*** **,** ***Dmp1*** **, and** ***Sos*** **t in 7-day osteogenic cultures of OPs.** The mRNA expression was analyzed by real-time RT-PCR. *Rankl*, receptor activator of nuclear factor-κB ligand; *Opg*, osteoprotegerin; *Dmp1*, dentin matrix acidic phosphoprotein 1; *Sost*, sclerostin. *p<0.05 vs. WT.
(PDF)

Figure S6 **Suppression of** ***Rankl*** **expression in response to the recovery of** ***Gli1*** **expression in** ***Gli1*** **^+/−^ BMSCs.** (**A**) Scheme of the experiment. *Gli*1^+/−^ BMSCs were cultured in osteogenic media supplemented with Smoothened agonist (SAG). Cells were infected with either Ax-*GFP* (control) or Ax-*GLI1-IRES-dsRed* on Day 4 and cultured for another 7 days. (**B**) mRNA expression of *Rankl* on days 0, 4, and 11. The mRNA expression was analyzed by real-time RT-PCR analyses. *p<0.05 vs. Ax-GFP.
(PDF)
