# Supplementary material for: Gli1 Haploinsufficiency Leads to Decreased Bone Mass with an Uncoupling of Bone Metabolism in Adult Mice
Source: PLoS One. 2014 Oct 14;9(10):e109597. doi: 10.1371/journal.pone.0109597 (PMC4196929; doi:10.1371/journal.pone.0109597)
Supplement: Figure S1 — Gross appearance of WT and Gli1 mutant male mice. (A) Gross appearance of WT, Gli1 +/−, and Gli1 −/− male mice at 8 weeks of age. (B) Comparison of postnatal growth between WT, Gli1 +/−, and Gli1 −/− male mice. Body weight was measured on the indicated dates after birth. *p<0.05 vs. WT or Gli1 +/−. (PDF) [file pone.0109597.s001.pdf]

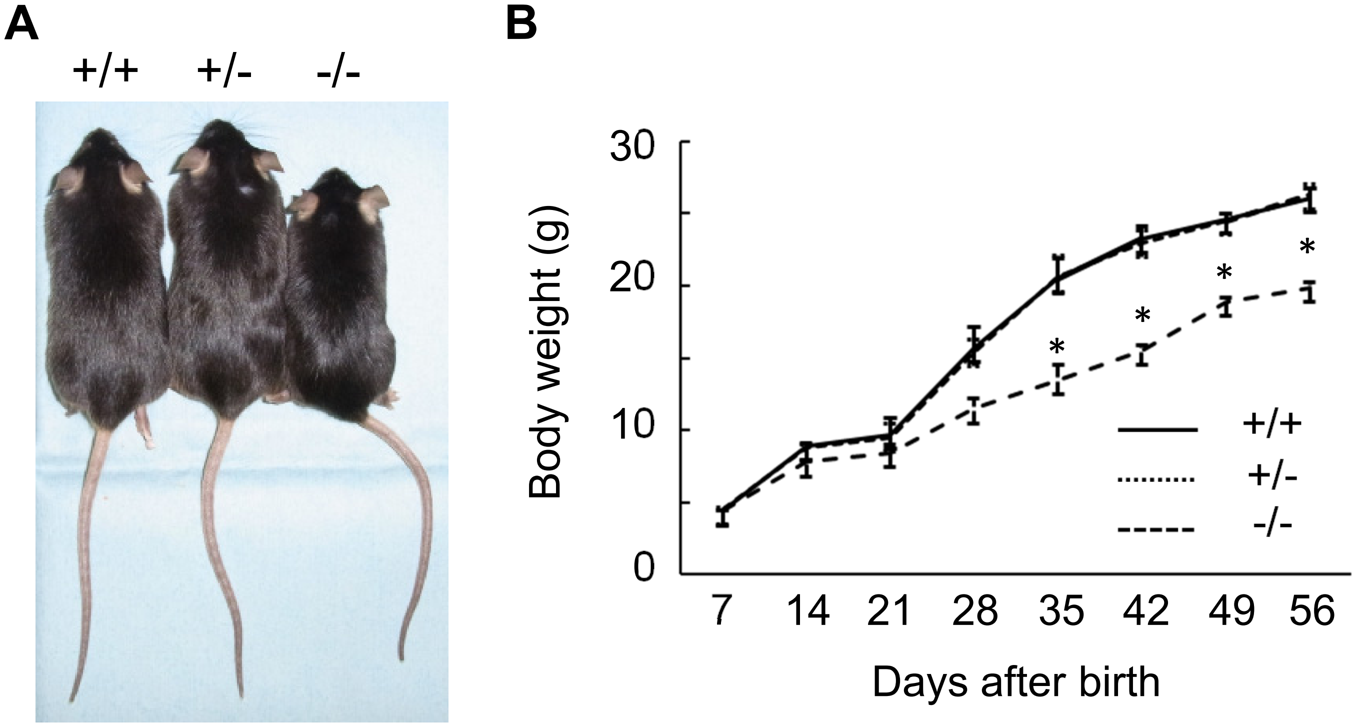

Figure S1 Gross appearance of WT and *Gli1* mutant male mice. (**A**) Gross appearance of WT, *Gli1*$^{+/-}$, and *Gli1*$^{-/-}$ male mice at 8 weeks of age. (**B**) Comparison of postnatal growth between WT, *Gli1*$^{+/-}$, and *Gli1*$^{-/-}$ male mice. Body weight was measured on the indicated dates after birth. *$p < 0.05$ vs. WT or *Gli1*$^{+/-}$.
