# Supplementary material for: Gli1 Haploinsufficiency Leads to Decreased Bone Mass with an Uncoupling of Bone Metabolism in Adult Mice
Source: PLoS One. 2014 Oct 14;9(10):e109597. doi: 10.1371/journal.pone.0109597 (PMC4196929; doi:10.1371/journal.pone.0109597)
Supplement: Figure S2 — Radiological analyses of long bones in female WT and Gli1 +/− mice. (A) 3D-micro-CT images of the distal femurs of representative 8-week-old WT and Gli1 +/− female mice. Sagittal sections, transverse sections, and 3D reconstruction images of the primary spongiosa are shown for each genotype. Bar, 1 mm. (B) Histomorphometric analyses of the 3D-micro-CT data in (A). BMD, bone mineral density; BV/TV, bone volume per tissue volume; Tb.Th, trabecular thickness; Tb.N, trabecular number parameters. Data are means ± SDs of five female mice per genotype. *p<0.05 vs. WT. (PDF) [file pone.0109597.s002.pdf]

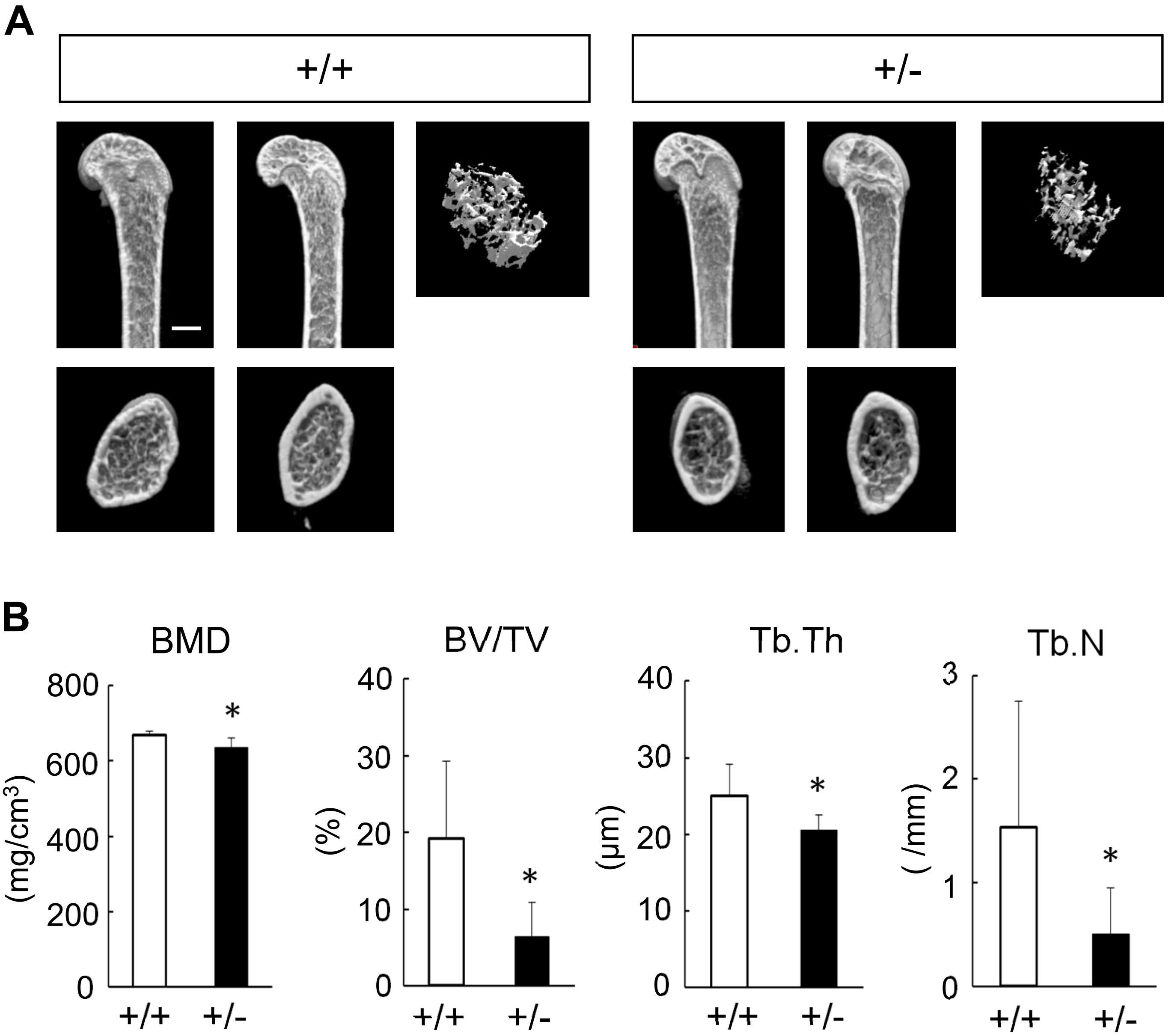

Figure S2 Radiological analyses of long bones in female WT and *Gli1*$^{+/-}$ mice. (**A**) 3D-micro-CT images of the distal femurs of representative 8-week-old WT and *Gli1*$^{+/-}$ female mice. Sagittal sections, transverse sections, and 3D reconstruction images of the primary spongiosa are shown for each genotype. Bar, 1 mm. (**B**) Histomorphometric analyses of the 3D-micro-CT data in (A). BMD, bone mineral density; BV/TV, bone volume per tissue volume; Tb.Th, trabecular thickness; Tb.N, trabecular number parameters. Data are means ± SDs of five female mice per genotype. *$p < 0.05$ vs. WT.
