# Supplementary material for: Gli1 Haploinsufficiency Leads to Decreased Bone Mass with an Uncoupling of Bone Metabolism in Adult Mice
Source: PLoS One. 2014 Oct 14;9(10):e109597. doi: 10.1371/journal.pone.0109597 (PMC4196929; doi:10.1371/journal.pone.0109597)
Supplement: Figure S3 — Radiological analyses of cortical bones in WT and Gli1 +/− mice. (A) 3D-micro-CT images of distal femurs of representative 8-week-old WT and Gli1 +/− male mice. Transverse sections of the primary spongiosa are shown for each genotype. Bar, 500 µm. (B) Histomorphometric analyses of 3D-micro-CT data in (A). Cv/Av, cortical bone volume per all bone volume; Cvt, cortical bone thickness; BMD, bone mineral density. Data are means ± SDs of ten male mice per genotype. (PDF) [file pone.0109597.s003.pdf]

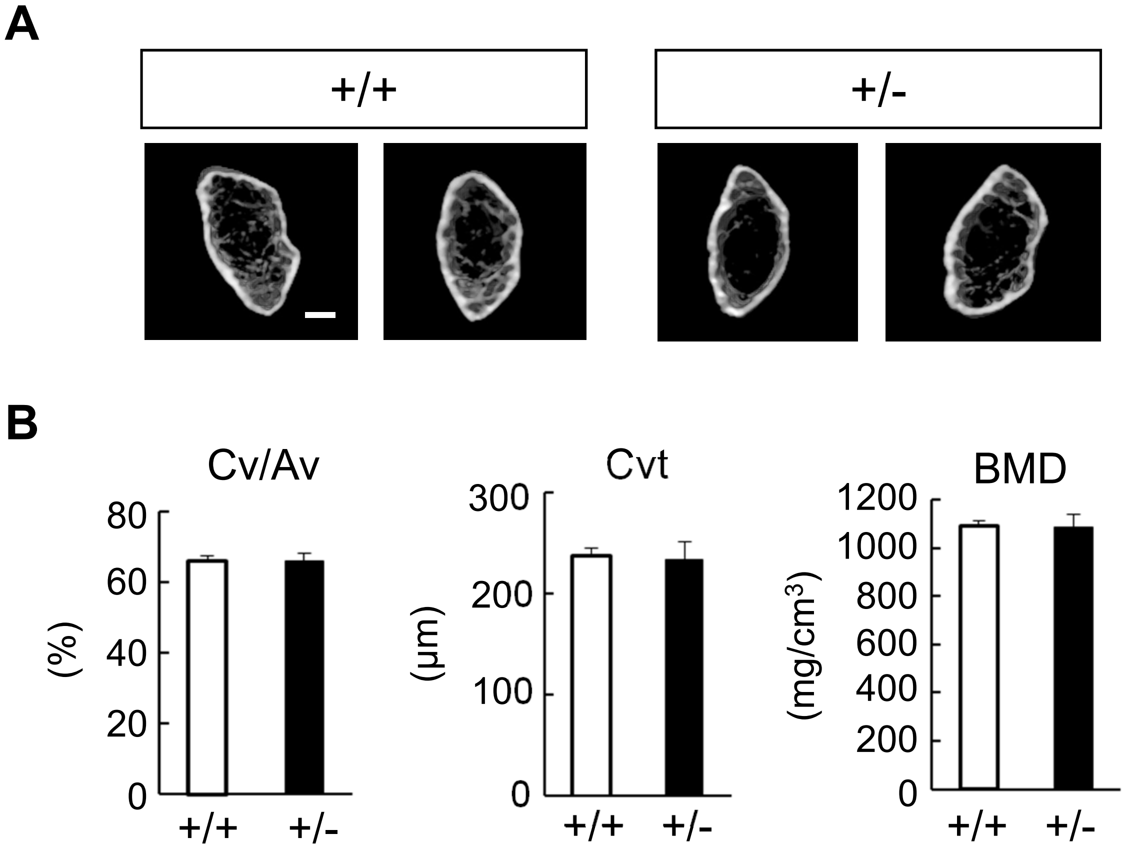

Figure S3 Radiological analyses of cortical bones in WT and *Gli1*$^{+/-}$ mice. (**A**) 3D-micro-CT images of distal femurs of representative 8-week-old WT and *Gli1*$^{+/-}$ male mice. Transverse sections of the primary spongiosa are shown for each genotype. Bar, 500 µm. (**B**) Histomorphometric analyses of 3D-micro-CT data in (A). Cv/Av, cortical bone volume per all bone volume; Cvt, cortical bone thickness; BMD, bone mineral density. Data are means ± SDs of ten male mice per genotype.
