# Supplementary material for: Gli1 Haploinsufficiency Leads to Decreased Bone Mass with an Uncoupling of Bone Metabolism in Adult Mice
Source: PLoS One. 2014 Oct 14;9(10):e109597. doi: 10.1371/journal.pone.0109597 (PMC4196929; doi:10.1371/journal.pone.0109597)
Supplement: Figure S4 — Construction of adenoviral vector expressing GLI1 . (A) Schematic representation of pAd/PL-DEST vectors expressing GLI1. (B) Luciferase reporter assay using the 8×3′-Gli BS-luc in combination with dsRed, Myc-GLI1, and the constructed adenoviral vector (GLI1-IRES-deRed). The luciferase assay was performed 48 hours after transfection in C3H10T1/2 cells. (C) Protein expression of GLI1 in C3H10T1/2 cells transfected with Myc-GLI1 or adenovirally transduced with GLI1-IRES-dsRed. (D) mRNA expression of Alp and Ibsp in C3H10T1/2 cells transfected with Myc-GLI1 or adenovirally transduced with GLI1-IRES-dsRed. (PDF) [file pone.0109597.s004.pdf]

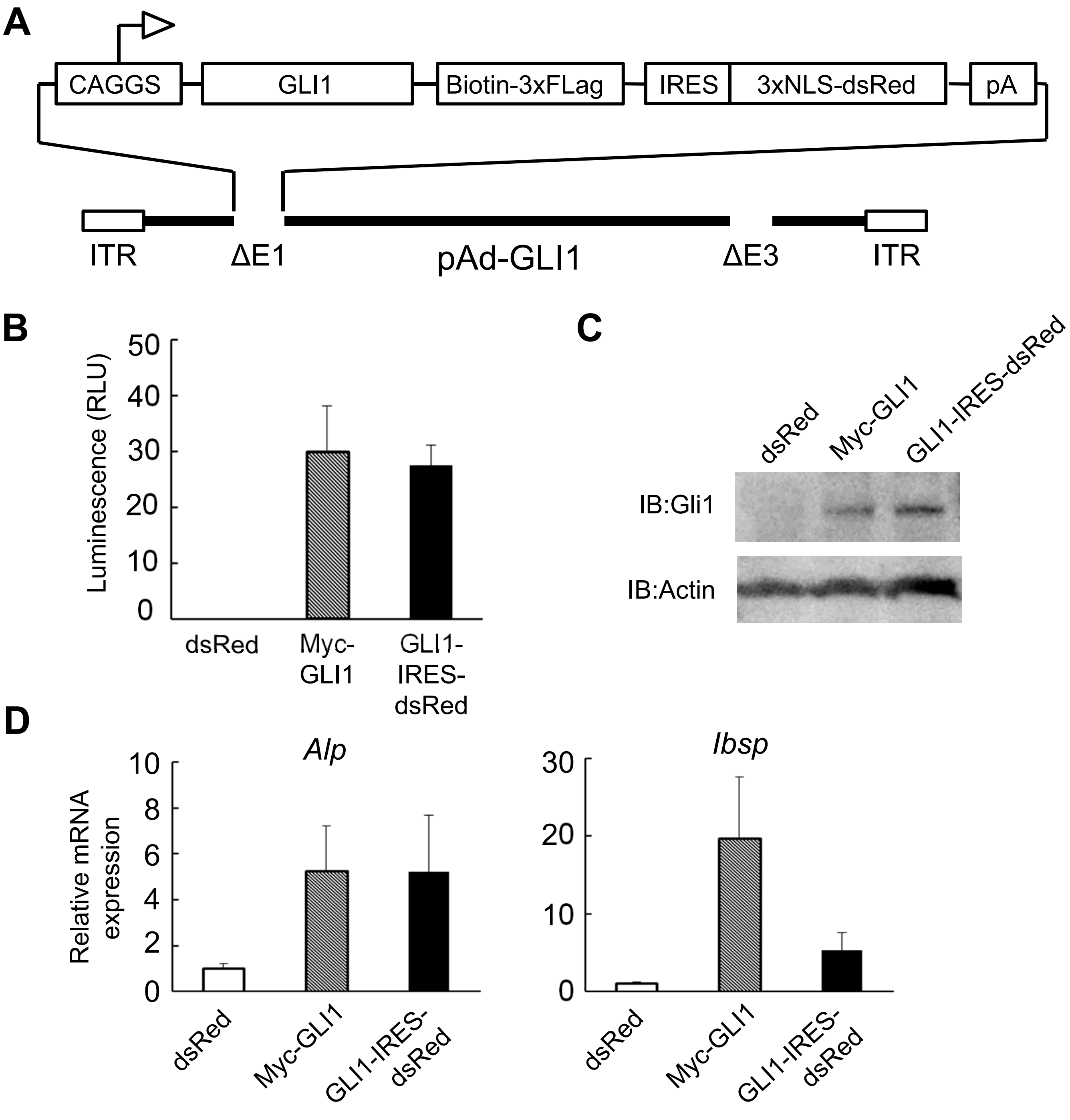

Figure S4 Construction of adenoviral vector expressing *GLI1*. (**A**) Schematic representation of pAd/PL-DEST vectors expressing *GLI1*. (**B**) Luciferase reporter assay using the 8 × 3'-Gli BS-luc in combination with *dsRed*, *Myc-GLI1*, and the constructed adenoviral vector (*GLI1-IRES-deRed*). The luciferase assay was performed 48 hours after transfection in C3H10T1/2 cells. (**C**) Protein expression of GLI1 in C3H10T1/2 cells transfected with *Myc-GLI1* or adenovirally transduced with *GLI1-IRES-dsRed*. (**D**) mRNA expression of *Alp* and *Ibsp* in C3H10T1/2 cells transfected with *Myc-GLI1* or adenovirally transduced with *GLI1-IRES-dsRed.*
