# Supplementary material for: Gli1 Haploinsufficiency Leads to Decreased Bone Mass with an Uncoupling of Bone Metabolism in Adult Mice
Source: PLoS One. 2014 Oct 14;9(10):e109597. doi: 10.1371/journal.pone.0109597 (PMC4196929; doi:10.1371/journal.pone.0109597)
Supplement: Figure S5 — mRNA expression of Rankl , Opg , Dmp1 , and Sos t in 7-day osteogenic cultures of OPs. The mRNA expression was analyzed by real-time RT-PCR. Rankl, receptor activator of nuclear factor-κB ligand; Opg, osteoprotegerin; Dmp1, dentin matrix acidic phosphoprotein 1; Sost, sclerostin. *p<0.05 vs. WT. (PDF) [file pone.0109597.s005.pdf]

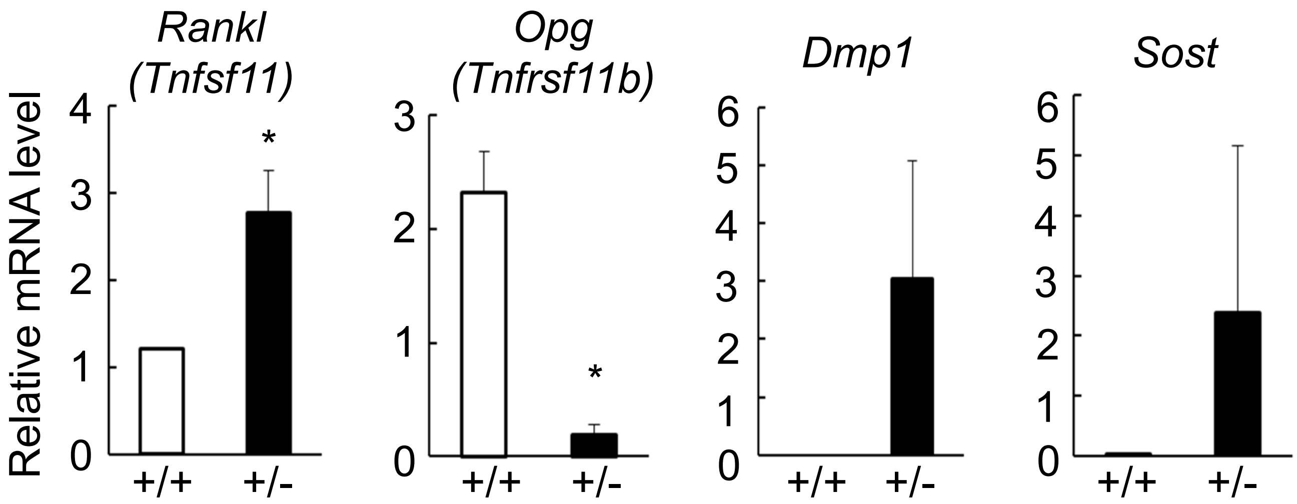

Figure S5 mRNA expression of *Rankl*, *Opg*, *Dmp1*, and *Sos*t in 7-day osteogenic cultures of OPs. The mRNA expression was analyzed by real-time RT-PCR. *Rankl,* receptor activator of nuclear factor-κB ligand; *Opg*, osteoprotegerin; *Dmp1*, dentin matrix acidic phosphoprotein 1; *Sost*, sclerostin. *p < 0.05 vs. WT.
