# Supplementary material for: Gli1 Haploinsufficiency Leads to Decreased Bone Mass with an Uncoupling of Bone Metabolism in Adult Mice
Source: PLoS One. 2014 Oct 14;9(10):e109597. doi: 10.1371/journal.pone.0109597 (PMC4196929; doi:10.1371/journal.pone.0109597)
Supplement: Figure S6 — Suppression of Rankl expression in response to the recovery of Gli1 expression in Gli1 +/− BMSCs. (A) Scheme of the experiment. Gli1+/− BMSCs were cultured in osteogenic media supplemented with Smoothened agonist (SAG). Cells were infected with either Ax-GFP (control) or Ax-GLI1-IRES-dsRed on Day 4 and cultured for another 7 days. (B) mRNA expression of Rankl on days 0, 4, and 11. The mRNA expression was analyzed by real-time RT-PCR analyses. *p<0.05 vs. Ax-GFP. (PDF) [file pone.0109597.s006.pdf]

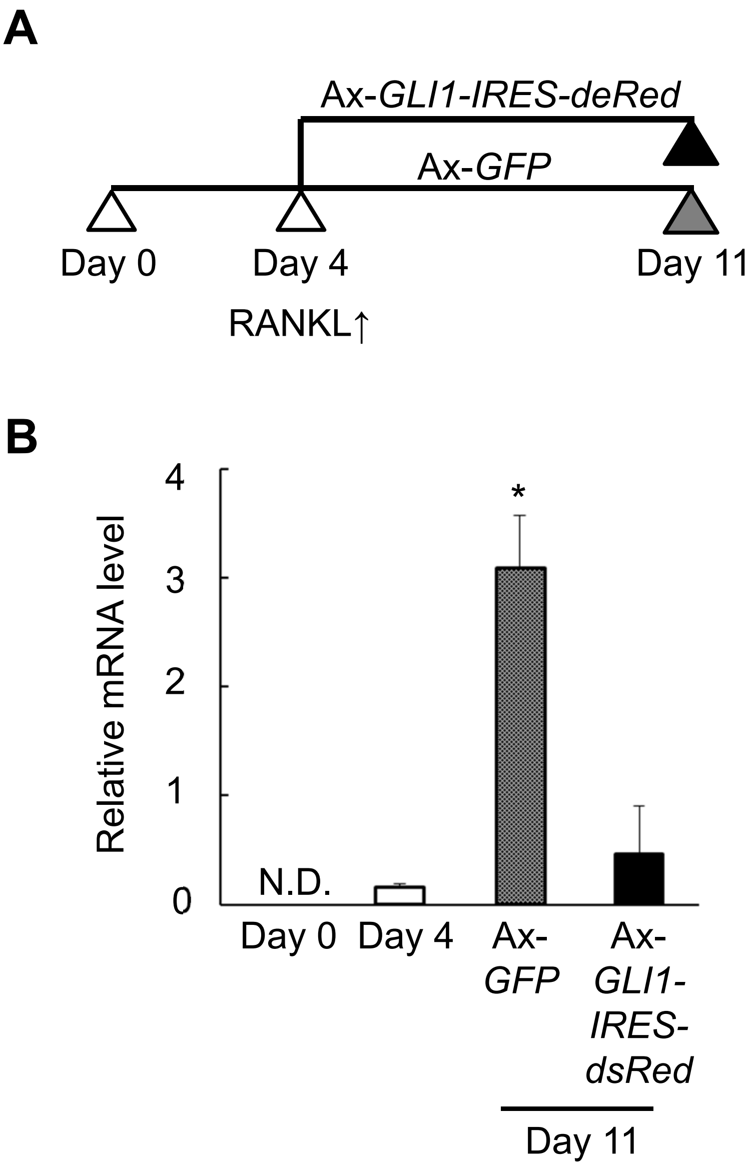

Figure S6 Suppression of *Rankl* expression in response to the recovery of *Gli1* expression in *Gli1*$^{+/-}$ BMSCs. (**A**) Scheme of the experiment. *Gli*1$^{+/-}$ BMSCs were cultured in osteogenic media supplemented with Smoothened agonist (SAG). Cells were infected with either Ax-*GFP* (control) or Ax-*GLI1-IRES-dsRed* on Day 4 and cultured for another 7 days. (**B**) mRNA expression of *Rankl* on days 0, 4, and 11. The mRNA expression was analyzed by real-time RT-PCR analyses. *$p < 0.05$ vs. Ax-*GFP*.
